# Naringin Inhibits Macrophage Foam Cell Formation by Regulating Lipid Homeostasis and Metabolic Phenotype

**DOI:** 10.3390/nu16091321

**Published:** 2024-04-28

**Authors:** Yan Liu, Xiaohan Tang, Hailong Yuan, Rong Gao

**Affiliations:** 1 Department of Pharmacy, Air Force Medical Center, PLA, Beijing 100142, China; liuyan990311@163.com (Y.L.); z17758133740@163.com (X.T.); yhlpharm@126.com (H.Y.); 2 School of Pharmacy, Anhui Medical University, Hefei 230032, China

**Keywords:** atherosclerosis, macrophage foam cell formation, naringin, metabolic reprogramming, inflammation

## Abstract

Imbalances in lipid uptake and efflux and inflammation are major contributors to foam cell formation, which is considered a therapeutic target to protect against atherosclerosis. Naringin, a citrus flavonoid abundant in citrus fruits, has been reported to exert an antiatherogenic function, but its pharmacological mechanism is unclear. Naringin treatment effectively inhibits foam cell formation in THP-1 and RAW264.7 macrophages. In this study, mechanically, naringin maintained lipid homeostasis within macrophages through downregulation of the key genes for lipid uptake (*MSR1* and *CD36*) and the upregulation of *ABCA1*, *ABCG1* and *SR-B1*, which are responsible for cholesterol efflux. Meanwhile, naringin significantly decreased the cholesterol synthesis-related genes and increased the genes involved in cholesterol metabolism. Subsequently, the results showed that ox-LDL-induced macrophage inflammatory responses were inhibited by naringin by reducing the proinflammatory cytokines IL-1β, IL-6 and TNF-α, and increasing the anti- inflammatory cytokine IL-10, which was further verified by the downregulation of pro-inflammatory and chemokine-related genes. Additionally, we found that naringin reprogrammed the metabolic phenotypes of macrophages by suppressing glycolysis and promoting lipid oxidation metabolism to restore macrophage phenotypes and functions. These results suggest that naringin is a potential drug for the treatment of AS as it inhibits macrophage foam cell formation by regulating metabolic phenotypes and inflammation.

## 1. Introduction

Macrophages are the most abundant component in atherosclerotic lesions and play a critical role in the formation, growth and rupture of arterial plaques by managing local lipids and inflammatory responses [1,2,3], making them elusive therapeutic targets of atherosclerosis (AS). It is generally acknowledged that monocytes are recruited to atherosclerotic lesions after cholesterol homeostasis imbalance and endothelial injury, and then differentiate into macrophages; eventually, these macrophages engulf modified lipoproteins such as oxidized low-density lipoprotein (ox-LDL), thereby forming foam cells. Lipid uptake, cholesterol esterification and cholesterol efflux are three interrelated biological processes that determine the formation of foam cells [4]. Macrophages take up ox-LDL through the cluster of differentiation 36 (CD36) and macrophage scavenger receptor 1 (MSR1) and uptake oxidized high-density lipoprotein (HDL) mainly via scavenger receptor B class type 1 (SR-B1), whereas they excrete cholesterol through the major transporters ATP-binding cassette protein A1 (ABCA1) and ATP-binding cassette protein G1 (ABCG1), which are directly regulated by liver X receptors (LXRs) [5]. Macrophage foam cells further secrete pro-inflammatory cytokines (IL-6, IL-1β and TNF-α) and adhesion molecules (monocyte chemoattractant protein 1, MCP-1; intercellular adhesion molecule 1, ICAM-1; and vascular cell adhesion molecule 1, VCAM-1) to induce the recruitment of monocytes/macrophages into the vascular walls [6,7], which further exacerbates lipid accumulation and contributes to necrotic core expansion [8,9]. Thus, targeting macrophage foam cell formation has been considered a promising strategy for treating and preventing AS.

Currently, natural functional ingredients with anti-AS effects have received increasing attention due to their relatively high safety, especially for preventive purposes, and can be used for long-term dietary interventions. Epidemiological studies have shown that fruit and vegetable intake is negatively correlated with the risk of AS, mainly due to their rich content of flavonoids, which have antioxidant and anti-inflammatory effects [10,11]. Naringin is a citrus flavonoid that is a very common dietary ingredient, especially abundant in citrus fruits, with antioxidant and anti-inflammatory effects [12]. It has also been observed in clinical studies that dietary naringin supplementation reduces human plasma LDL-C and apolipoprotein B levels [13]. In addition, naringin has a good safety profile, with multiple cell line studies having shown the low cytotoxicity of naringin, and a single oral administration of 2 g of naringin has no adverse effects on patients [14]. Hence, naringin offers great potential as a preventive and therapeutic agent for AS. However, the role of naringin in foam cell formation and its underlying molecular mechanism remain unclear.

In this study, we investigated the effects of naringin on foam cell formation in THP-1 and RAW264.7 in vitro models to determine the optimal condition that naringin exhibited. Secondly, the regulatory effects of naringin on key molecules involved in lipid uptake, cholesterol metabolism and cholesterol efflux in macrophages, as well as inflammation, were further evaluated. Finally, the transcriptional regulation and metabolic reprogramming mechanisms involved in this progress were explored to provide new possibilities for AS prevention and treatment strategies.

## 2. Materials and Methods

### 2.1. Chemicals

Naringin (≥98%) was obtained from Baoji Herbest Bio-Tech Co., Ltd. (Baoji, China). THP-1 and RAW264.7 were obtained from Procell Life Science & Technology Co., Ltd. (Wuhan, China). RPMI 1640 medium, fetal bovine serum (FBS), penicillin mixture and 0.25% Trypsin-EDTA were purchased from Gibco (Carlsbad, CA, USA). 2-Acetoxy-1-methoxypropane (PMA) and dimethyl sulfoxide (DMSO) were obtained from Sigma-Aldrich (St. Louis, MO, USA). A cell counting kit-8 (CCK-8) was purchased from Elabscience Biotechnology Co., Ltd. (Wuhan, China). ox-LDL and Dil-ox-LDL were purchased from Yiyuan Biotechnology (Guangzhou, China). A free cholesterol (FC) content assay kit and a total cholesterol (TC) content assay kit were obtained from Solarbio Technology Co., Ltd. (Beijing, China). IL-1β, IL-6, IL-10, TNF-α and MCP-1 ELISA kits were obtained from BOSTER Biological Technology Co., Ltd. (Wuhan, China). A cholesterol efflux assay kit was purchased from Abcam (Cambridge, UK). Antibodies were purchased from ABclonal Technology Co., Ltd. (Wuhan, China).

### 2.2. Cell Culture

THP-1 cells were maintained in 1640 medium with 10% FBS and 0.05 mM 2-Hydroxy-1-ethanethiol at 37 °C in a 5% CO_2_ humidified air incubator. RAW264.7 cells were cultured in 1640 medium containing 10% FBS. THP-1 macrophages were obtained by inducing the THP-1 monocytes with 100 ng/mL PMA for 48 h. The obtained THP-1 and RAW264.7 macrophages were incubated with 50 μg/mL ox-LDL for 48 h to construct the macrophage foaming model. The cells were passaged every 2 or 3 days.

### 2.3. Cell Viability Assay

Cell viability was measured by the CCK-8 assay according to the manufacturer’s instructions. After seeding THP-1 and RAW264.7 macrophages into a 96-well plate at a concentration of 5 × 10^5^ cells/mL, the cells were exposed to different concentrations of naringin (0, 2, 4, 8, 16, 32, 64, 128 and 256 µg/mL) diluted in culture medium for 48 h at 37 °C. Then, the absorption at 450 nm was measured after 2 h of incubation with a 10 µL CCK-8 solution in each well.

### 2.4. Oil Red O Staining

THP-1 and RAW264.7 cells were seeded into a 6-well plate at a concentration of 2.5 × 10^5^ cells/mL. The THP-1 cells were treated with 100 ng/mL PMA for 48 h. Then, the THP-1 and RAW264.7 cells were incubated with different stimuli (Control group: culture medium; ox-LDL group: 50 μg/mL ox-LDL; Naringin groups: 50 μg/mL ox-LDL+ 4, 8, 16 and 32 µg/mL naringin) for 48 h at 37 °C to study the effects of naringin on the lipid content of macrophages. After oil red O staining, the effect of lipid droplet staining was observed under a light microscope, followed by the addition of 200 µL of isopropanol and decolorizing extraction for 30 min. The absorbance was measured at 490 nm to further determine the optimal concentration of naringin.

### 2.5. Effects of Naringin on Cholesterol Levels in Macrophages

THP-1 and RAW264.7 macrophages were incubated with different stimuli (Control group: culture medium; ox-LDL group: 50 μg/mL ox-LDL; Naringin groups: 50 μg/mL ox-LDL+ 32 µg/mL naringin) for 48 h at 37 °C to study the effects of naringin on the cholesterol levels in macrophages. Free and total cholesterol levels in macrophages were measured according to the free and total cholesterol content assay kit instructions and we calculated the ratio of cholesterol ester to total cholesterol (CE/TC). The working solutions were added to the cell extracts and then allowed to stand for 15 min at 37 °C. The absorbance at 500 nm was measured after completion of the reaction.

### 2.6. Dil-ox-LDL Uptake Assay

THP-1 macrophages were incubated with 50 μg/mL Dil-ox-LDL or/and 32 μg/mL naringin for 4 h at 37 °C. The uptake of Dil-ox-LDL was examined using fluorescence microscopy, and fluorescence intensity was analyzed using Image Pro Plus 6.0 software (Ex/Em = 554/571 nm).

### 2.7. Cholesterol Efflux Assay

The cholesterol efflux of cells was detected by the cholesterol efflux assay kit in accordance with the instructions. THP-1 macrophages were inoculated into 96-well plates at a cell density of 5 × 10^5^ cells/mL, serum-free medium containing labeling medium was added, and the cells were incubated for 1 h. A total of 100 µL of equilibrium medium was added to each well and incubated at 37 °C overnight. The equilibrium medium was aspirated, and after 24 h of incubation with or without 32 µg/mL naringin, the supernatant of each well was transferred to a 96-well plate, 100 µL of lysate was added to the cells, the cells were incubated on a shaker for 30 min and the fluorescence intensity was detected by a microplate reader (Ex/Em = 485/523 nm).

(1) $$\%\;Cholesterol\;Efflux=\frac{RFU\;of\;Supernatant}{RFU\;of\;Cell\;Lysate+RFU\;of\;Supernatant}\times 100$$

### 2.8. Cytokine ELISA Assays

THP-1cells were seeded into 100 mm plates at a concentration of 2.5 × 105 cells/mL and treated with 100 ng/mL PMA for 48 h. After 48 h of incubation with different stimuli, cells were then cultured in serum-free medium for another 24 h. The culture medium was collected for the ELISA assays. The contents of cytokines (IL-1β, IL-6, IL-10 MCP-1 and TNF-α) in the medium and serum of mice were measured according to the instructions of the kits.

### 2.9. Western Blot

After treatment with naringin under the optimal conditions, the THP-1 macrophages were lysed with RIPA lysate to extract total cellular proteins. Total protein content was quantified using the BCA protein concentration assay kit, and protein samples were electrophoresed in 10% SDS-PAGE. Then, the separated protein gel was transferred to a PVDF membrane. The internal reference was β-Actin.

### 2.10. Real-Time qPCR Analysis

After treatment with naringin under the optimal conditions, total cellular RNA was extracted using Trizol reagent (Solarbio Technology Co., Ltd., Beijing, China). The cDNA was reverse transcribed according to the PrimeScript™ RT reagent kit instructions. Then, the cDNA fragments were amplified, and the fluorescence of each gene was detected using a CFX96 Deep Well^TM^ Real-Time System (Bio-Rad Laboratories, Hercules, CA, USA). PCR cycling conditions: 95 °C for 1 min, 95 °C for 10 s, 60 °C for 10 s, 72 °C for 15 s; 40 cycles; fluorescence was collected at 72 °C. The relative expression of each gene was calculated using the 2^−ΔΔCt^ method, and the internal reference gene was β-Actin. Primers are shown in Table 1.

### 2.11. RNA Sequencing (RNA-seq) and Data Analysis

RNA was extracted from the ox-LDL-induced THP-1 macrophages treated with or without naringin, and then analyzed as previously described [15]. The differentially expressed genes (DEGs) were identified by using the DESeq2 R package (1.20.0). The genes with padj ≤ 0.05 and |log2(foldchange)| ≥ 1 were considered significantly different. Gene ontology (GO) and Kyoto encyclopedia of genes and genomes (KEGG) pathway enrichment analyses of differentially expressed genes were performed using the clusterProfiler R package (4.2.0).

### 2.12. Untargeted Metabolomics Analysis

The ox-LDL-induced THP-1 macrophages (2.5 × 10^6^ cells per sample) treated with or without naringin were extracted with 300 μL prechilled 80% methanol for 6 min. After centrifugation at 12,000× *g* for 15 min, 100 µL supernatant of each sample was collected for further analysis. Untargeted metabolomics analysis was performed using a Vanquish UHPLC system coupled with an Orbitrap Q ExactiveTMHF-X mass spectrometer (Thermo Fisher, Dreieich, Germany) in ESI+/ESI− modes. The gradient mobile phase was composed of water with 0.1% formic acid (A) and methanol (B) for ESI+ mode, and 5 mM ammonium acetate (pH 9.0) (A) and methanol (B) for ESI-mode. The solvent gradient was set as follows: 2% B, 1.5 min; 2–85% B, 3 min; 85% to 100% B, 10 min; 100% to 2% B, 10.1 min; 2% B, 12 min.

The acquired data were processed by the Compound Discoverer 3.1 (CD3.1, ThermoFisher, Karlsruhe, Germany). Principal component analysis (PCA) and partial least squares discriminant analysis (PLS-DA) were carried out utilizing SIMCA-p version 13.0. The metabolites with variable importance in projection (VIP) values > 1 and *p*-values < 0.05 were considered to be differential metabolites.

### 2.13. Statistical Analysis

All data were expressed as means ± standard deviation (SD). One-way ANOVA was used to evaluate differences among multiple groups, and Student’s *t*-tests were used to compare two groups. All data were analyzed using GraphPad Prism 9.0, R 4.2.0 and Python 2.7.6. *p* < 0.05 was considered to be statistically significant.

## 3. Results

### 3.1. Naringin Inhibits the Foam Cell Formation of Macrophages Induced by ox-LDL

**Figure 1:**
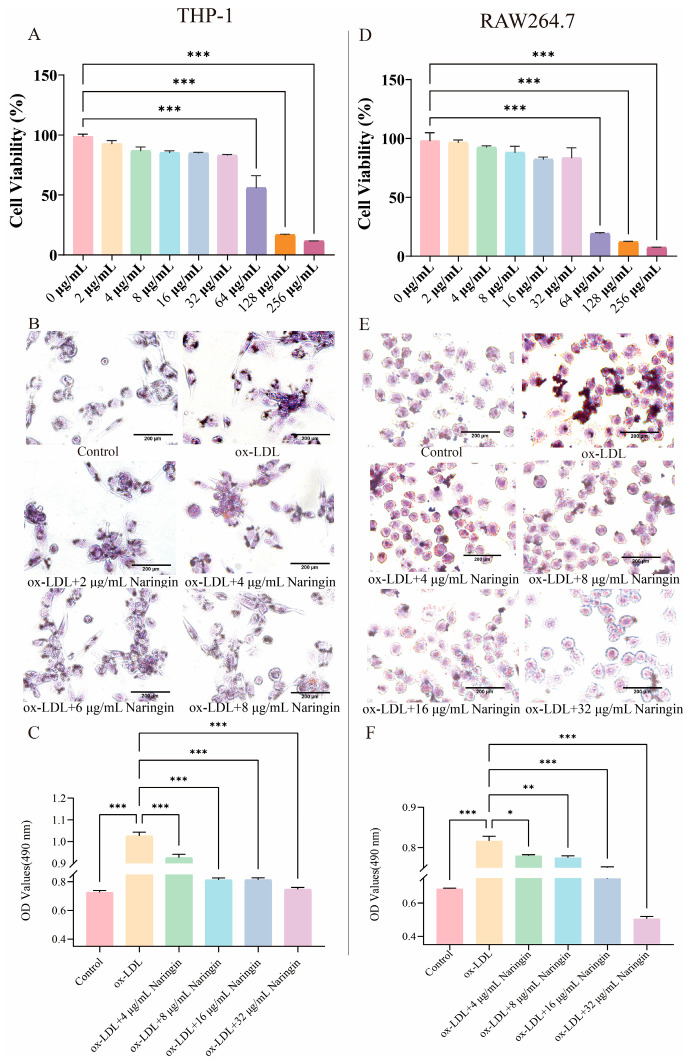
The effects of naringin on macrophage foam cell formation. (**A**) Effects of naringin on THP-1 cell viability. THP-1 cells were exposed to various concentrations of naringin (0, 2, 4, 8, 16, 32, 64, 128 and 256 μg/mL) for 48 h. (**B**) Representative images of oil red O staining of lipid accumulation in ox-LDL-induced THP-1 macrophages with and without naringin treatment (0, 2, 4, 8, 16 and 32 μg/mL). Scale bar = 200 μm. (**C**) Effects of naringin on relative contents of intracellular oil red measured by colorimetric method in THP-1 macrophages. (**D**) Effects of naringin on RAW264.7 cell viability. RAW264.7 cells were exposed to various concentrations of naringin (0, 2, 4, 8, 16, 32, 64, 128 and 256 μg/mL) for 48 h. (**E**) Representative images of oil red O staining of lipid accumulation in ox-LDL-induced RAW264.7 macrophages with and without naringin treatment (0, 2, 4, 8, 16 and 32 μg/mL). Scale bar = 200 μm. (**F**) Effects of naringin on relative contents of intracellular oil red measured by colorimetric method in RAW264.7 macrophages. Data are represented as means ± SD (n = 3). * *p* < 0.05, ** *p* < 0.01 and *** *p* < 0.001.

The THP-1 monocytes were rounded suspension cells, while the THP-1 macrophages obtained after inhibition with 100 ng/mL PMA for 48 h were irregular walled cells with pseudopods. THP-1 and RAW264.7 macrophages were treated for 48 h with 50 g/mL ox-LDL to construct a macrophage foam cell model. The cell viability assay results showed that naringin did not significantly affect the cell viability in the concentration range of 0–32 µg/mL in both THP-1 (Figure 1A) and RAW264.7 macrophages (Figure 1D). Thus, we further investigated the effects of naringin on macrophage foaming at concentrations of less than 32 µg/mL.

The oil red O staining results showed that ox-LDL induction significantly increased intracellular lipid droplets, while naringin could reduce intracellular lipid aggregation and inhibit foam cell formation in THP-1 (Figure 1B,C) and RAW264.7 cells (Figure 1E,F), in a concentration-dependent manner. The optimal inhibitory effect was achieved when the naringin concentration was 32 µg/mL. Therefore, 32 µg/mL of naringin was chosen as the optimal concentration in subsequent experiments.

### 3.2. Naringin Inhibits the Synthesis of Cholesterol Esters

**Figure 2:**
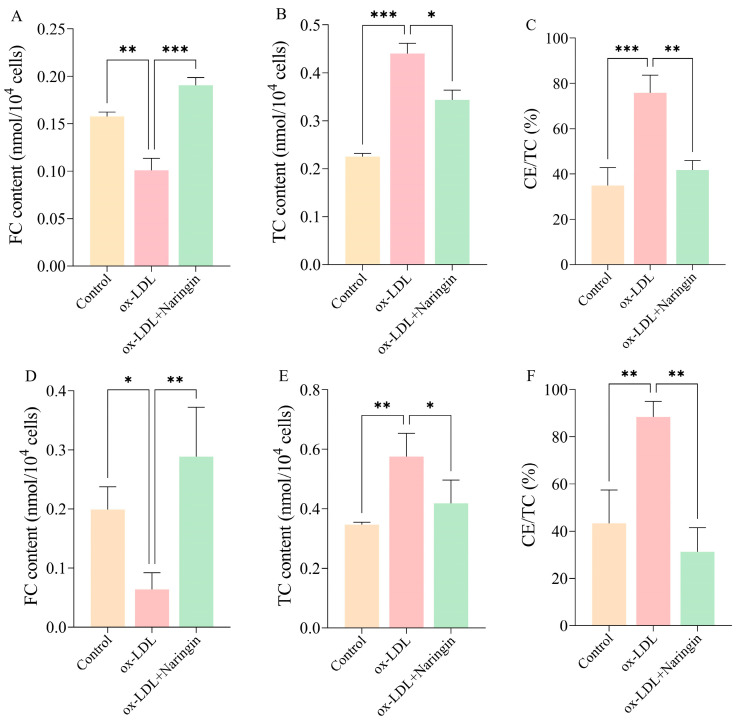
The effects of 32 µg/mL naringin on the contents of TC, FC and CE/TC in THP-1 and RAW264.7 macrophages. (**A**) The content of TC in THP-1 macrophages. (**B**) The content of TC in THP-1 macrophages. (**C**) The level of CE/TC in THP-1 macrophages. (**D**) The content of FC in RAW264.7 macrophages. (**E**) The content of TC in RAW264.7 macrophages. (**F**) The level of CE/TC in RAW264.7 macrophages. * *p* < 0.05, ** *p* < 0.01 and *** *p* < 0.001.

After the treatment with 32 µg/mL naringin for 48 h, we detected the contents of TC, FC and CE/TC in THP-1 and RAW264.7 macrophages to assess the effects of the synthesis of cholesterol esters (Figure 2). The results showed that the intracellular FC was significantly decreased while TC and CE/TC were increased in THP-1 macrophages after ox-LDL incubation (Figure 2A–C), and showed a consistent trend of change in RAW264.7 macrophages (Figure 2D–F). Compared with the ox-LDL group, naringin significantly reduced total cholesterol levels, increased the free cholesterol content, and inhibited cholesteryl ester aggregation in THP-1 (Figure 2A–C) and RAW.264.7 macrophages (Figure 2D–F). This suggests that naringin may exert its anti-AS effects by inhibiting cholesterol synthesis in macrophages.

### 3.3. Naringin Inhibits ox-LDL Uptake and Promotes Cholesterol Efflux in THP-1 Macrophages

**Figure 3:**
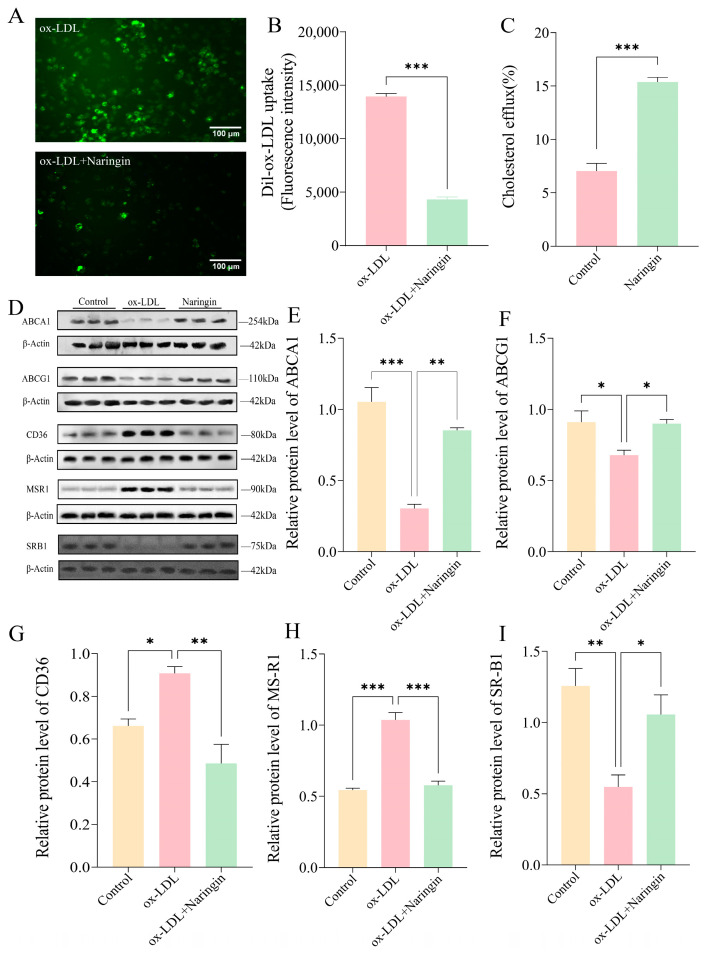
The effects of 32 µg/mL naringin on lipoprotein uptake and cholesterol efflux. (**A**) Representative fluorescent staining images of the Dil-ox-LDL uptake in macrophages with and without naringin treatment. The Dil-oxLDL signals are shown in green. Scale bar = 200 μm. (**B**) The mean fluorescence intensity of Dil-oxLDL uptake in THP-1 macrophages (n = 3). (**C**) Effects of naringin on cholesterol efflux in THP-1 macrophages (n = 3). (**D**–**I**) Effects of naringin on the protein expression levels of lipid uptake- and cholesterol efflux-related receptors in THP-1 macrophage. * *p* < 0.05, ** *p* < 0.01 and *** *p* < 0.001.

Macrophage foam cells play a crucial role in the atherosclerotic process, which is mainly caused by an imbalance in the biological effects of lipid uptake, esterification and cholesterol efflux [15]. Therefore, we evaluated the influence of naringin on the uptake of Dil-ox-LDL and the efflux of labeled cholesterol in THP-1 macrophages. The results showed that naringin significantly inhibited the uptake of Dil-ox-LDL in THP-1 macrophages (Figure 3A,B). Meanwhile, the cholesterol efflux in THP-1 macrophages increased more than two-fold after naringin treatment (Figure 3C).

**Figure 4:**
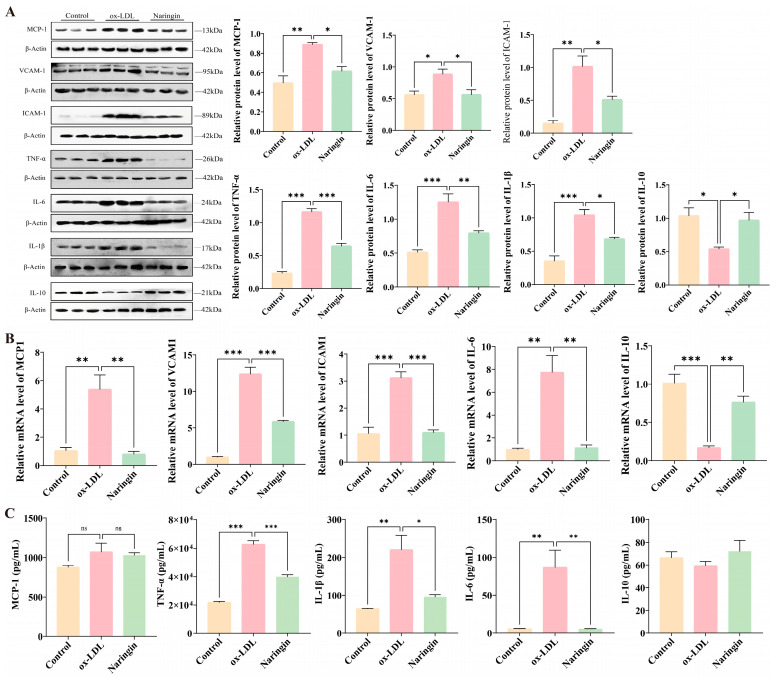
The effects of naringin on cell adhesion and inflammatory responses. (**A**) The protein expression of MCP-1, VCAM-1, ICAM-1, TNF-α, IL-6, IL-1β and IL-10. (**B**) The mRNA expression of MCP-1, VCAM-1, ICAM-1, IL-6 and IL-10. (**C**) The effects of naringin on cytokine secretion into the medium. * *p* < 0.05, ** *p* < 0.01 and *** *p* < 0.001 (n = 3).

Subsequently, we measured the expression of the major scavenger receptors (SRs) and cholesterol transporters in THP-1 macrophages by Western blot, including the lipid uptake-associated receptors CD36 and MSR1, and cholesterol efflux-related receptors SR-B1, ABCA1 and ABCG1. The results illustrated that naringin significantly downregulated the protein expression of CD36 and MSR1, whereas it upregulated the expression of SR-B1, ABCA1 and ABCG1 (Figure 4D–I). Collectively, these findings demonstrated that naringin could suppress macrophage foam cell formation by inhibiting lipoprotein uptake and promoting cholesterol efflux.

### 3.4. Naringin Relieves Cell Adhesion and Inflammatory Responses in THP-1 Macrophages

**Figure 5:**
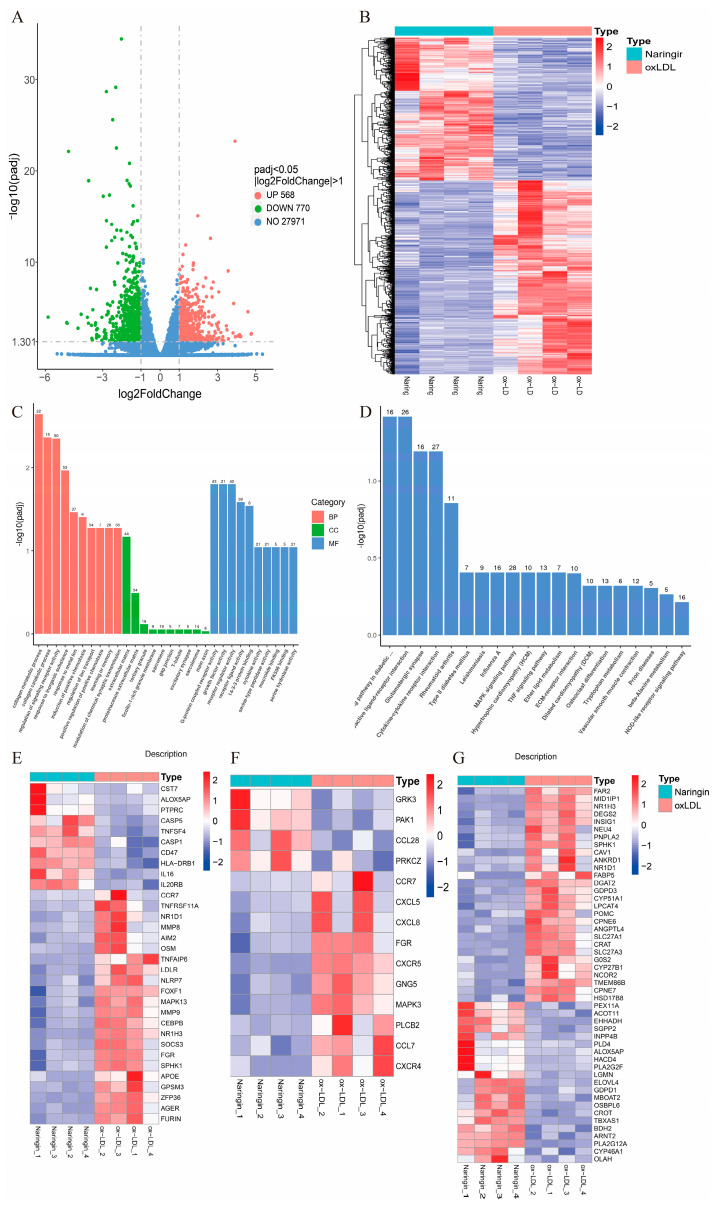
Effects of naringin on the transcriptome of ox-LDL-stimulated macrophages. (**A**) Volcano plot to identify the DEGs in ox-LDL-stimulated macrophages with and without naringin treatment. (**B**) Heatmap plot of DEG expression in ox-LDL and naringin groups. (**C**) GO enrichment analysis of the DEGs. BP, biological process; CC, cell component; MF, molecular function. (**D**) KEGG pathways of the DEGs. The numbers on the bars represent the number of genes involved. The -log10 (padj) values are shown on the *y*-axis. (**E**) The expression of the inflammatory and chemokine-related DEGs in the ox-LDL and naringin groups. (**F**) The expression of glycolysis-related DEGs in the ox-LDL and naringin groups. (**G**) The expression of lipid metabolism-related DEGs in the ox-LDL and naringin groups. The ox-LDL and naringin groups represent ox-LDL-stimulated macrophages without and without naringin treatment, respectively. n = 4.

It is recognized that inflammation plays a pivotal role in the formation of foaming cells, which, in turn, promotes monocyte migration and adhesion to endothelial cells, and further exacerbates AS progression [16]. Therefore, the effect of naringin on the inflammatory mediators in THP-1 macrophages induced by ox-LDL was investigated. The results showed that naringin could markedly reduce the protein expression of MCP-1, VCAM-1 and ICAM-1, which were appreciably induced by ox-LDL (Figure 4A). Moreover, naringin downregulated the protein level of the proinflammatory cytokines IL-6, IL-1β and TNF-α, and upregulated the expression of the anti-inflammatory cytokine IL-10 (Figure 4A). The qPCR results further verified naringin’s anti-adhesion and anti-inflammatory properties (Figure 5B). In addition, we measured cytokine secretion into the medium, and found that naringin considerably inhibited the secretion of IL-1β, IL-6 and TNF-α which was induced by ox-LDL, whereas it had no effect on MCP-1 and IL-10 levels in the medium (Figure 4C).

### 3.5. Naringin Inhibits the Expression of Pro-Inflammatory Macrophage Phenotype-Related Genes

**Figure 6:**
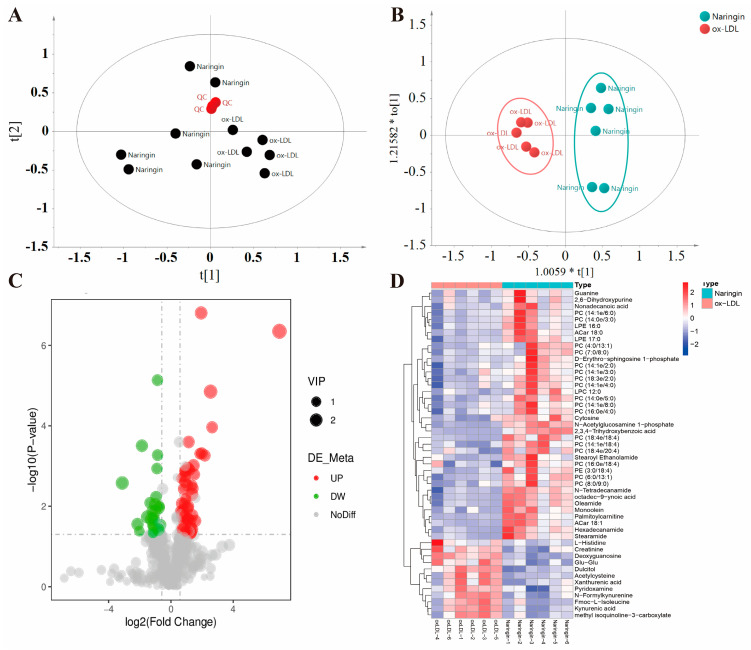

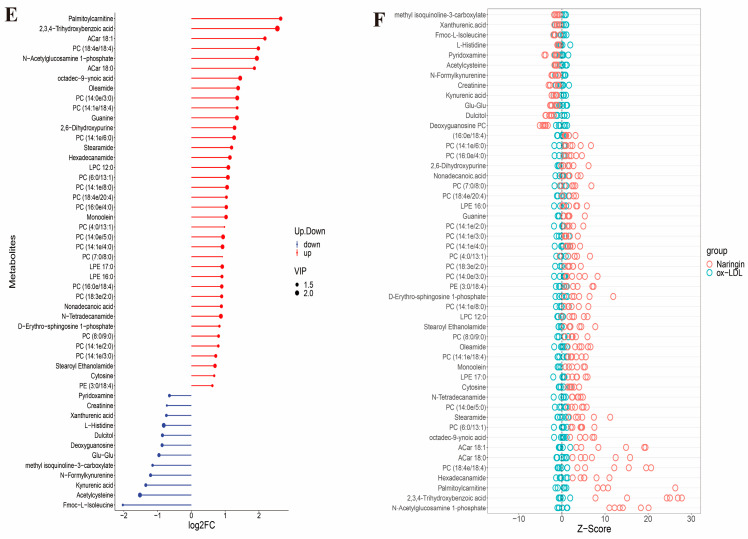
Effects of naringin on the metabolic phenotypes of ox-LDL-stimulated macrophages in positive mode. (**A**) PCA score plot for all the QC (red), ox-LDL (black) and naringin (black) samples. (**B**) OPLS-DA score plot for the ox-LDL (red) and naringin (blue) samples. (**C**) Volcano plot to identify the DEMs in ox-LDL-stimulated macrophages with and without naringin treatment. (**D**) Heatmap plot of the DEMs in the ox-LDL and naringin groups. (**E**) Stem plot of the DEMs. (**F**) Z score plot of the DEMs in the ox-LDL and naringin groups.

In order to identify the molecular mechanisms by which naringin regulates macrophage foaming, RNA-seq was performed on the ox-LDL-stimulated THP-1 foam cell model with and without naringin treatment. Consequently, 1338 DEGs were identified after the treatment of naringin, including 568 upregulated genes and 770 downregulated genes (Figure 6A,B). Then, GO and KEGG enrichment analyses were conducted to reveal the potential biological functions of the DEGs. GO term analysis revealed the DEGs were highly enriched in the collagen metabolic process, the regulation of signaling receptor activity, the response to inorganic substances, the response to metal ions, the induction of positive chemotaxis, the regulation of ion transport, the positive regulation of positive chemotaxis, learning or memory and the modulation of chemical synaptic transmission in the biological process (BP) category; the extracellular matrix, proteinaceous extracellular matrix and tertiary granule in the cell component (CC) category; and G-protein-coupled receptor activity, growth factor activity, receptor regulator activity, cytokine activity, serine-type peptidase activity and serine hydrolase activity in the molecular function (MF) category (Figure 6C). Moreover, the KEGG analysis showed that the DEGs were linked to glutamatergic synapses, cytokine–cytokine receptor interaction, the MAPK signaling pathway, the TNF signaling pathway, tryptophan metabolism, vascular smooth muscle contraction and the NOD-like receptor signaling pathway (Figure 5D).

Since AS is closely associated with abnormal cell metabolism and inflammation, we further investigated the regulatory effect of naringin on 28 inflammatory response related genes, 16 glycolysis-related genes and 49 lipid metabolism-related genes which were obtained from the intersection of the DEGs and inflammation, chemokines and lipid metabolism-related gene sets from the Molecular Signature Database (MsigDB). The results indicated that naringin significantly downregulated most of the inflammatory and chemokine-related genes (Figure 6E). Inflammatory macrophages have been reported to exhibit an increase in glycolytic activity and further promote inflammatory cytokine secretion [17,18]. As expected, naringin significantly reduced most glycolysis-related genes, especially the key genes in glycolysis hexokinase 3 (*HK3*) and 6-phosphofructo-2-kinase/fructose-2,6-biphosphatase 4 (*PFKFB4*) (Figure 6F). In addition, we further found that naringin significantly downregulated the DEGs associated with cholesterol synthesis (*NR1H3*; *ANGPTL4*, *INSIG1*, *CYP51A1* and *CYP27A1*), fatty acid transporter (*CAV1*, *SLC27A3*, *FABP5* and *CRAT*), triglyceride or glycerophospholipid biosynthesis (*GDPD3*, *G0S2*, *DGAT2*, *SPHK1* and *LPCAT4*) and fatty acid biosynthesis (*DGAT2*, *MID1IP1* and *DEGS2*), while it upregulated cholesterol metabolism (*CYP46A1* and *OSBPL6*), glycerophospholipid catabolism (*PLA2G2F*, *INPP4B*, *SGPP2*, *PLA2G12A* and *GDPD1*) and fatty acid catabolism (*ALOX5AP*, *HACD4*, *EHHADH*, *CROT*, *BDH2* and *ACOT11*)-related DEGs (Figure 5F).

Collectively, naringin inhibited the foaming formation of macrophages via the alleviation of inflammation by downregulating pro-inflammatory and glycolysis-related genes and regulating lipid metabolism-related genes.

### 3.6. Naringin Reprogrammed the Metabolic Phenotypes of Macrophages

**Figure 7:**
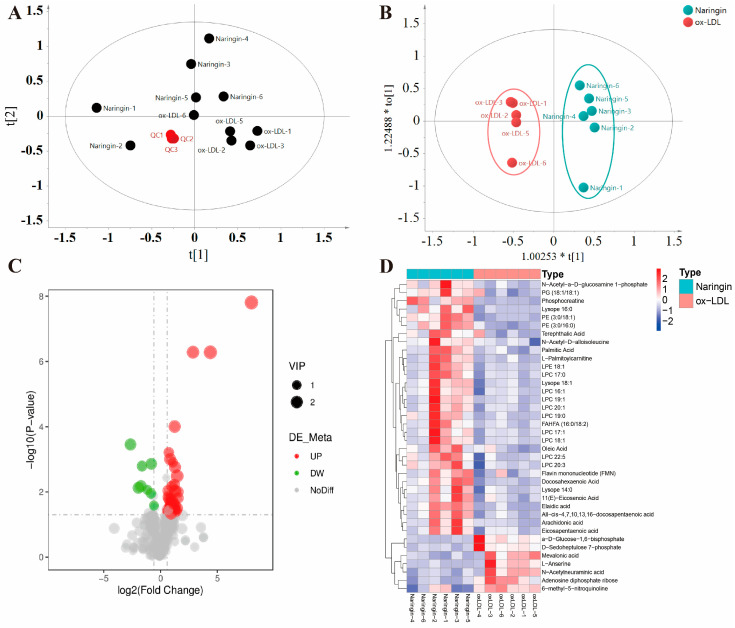

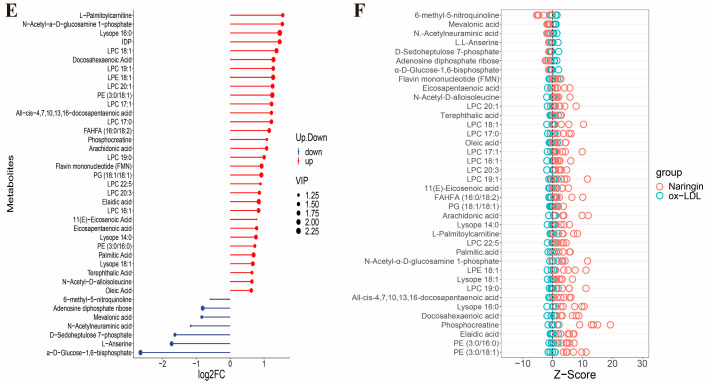
Effects of naringin on the metabolic phenotypes of ox-LDL-stimulated macrophages in negative mode. (**A**) PCA score plot for all the QC (red), ox-LDL (black) and naringin (black) samples. (**B**) OPLS-DA score plot for the ox-LDL (red) and naringin (blue) samples. (**C**) Volcano plot to identify the DEMs in ox-LDL-stimulated macrophages with and without naringin treatment. (**D**) Heatmap plot of the DEMs in the ox-LDL and naringin groups. (**E**) Stem plot of the DEMs. (**F**) Z score plot of the DEMs in the ox-LDL and naringin groups.

**Figure 8:**
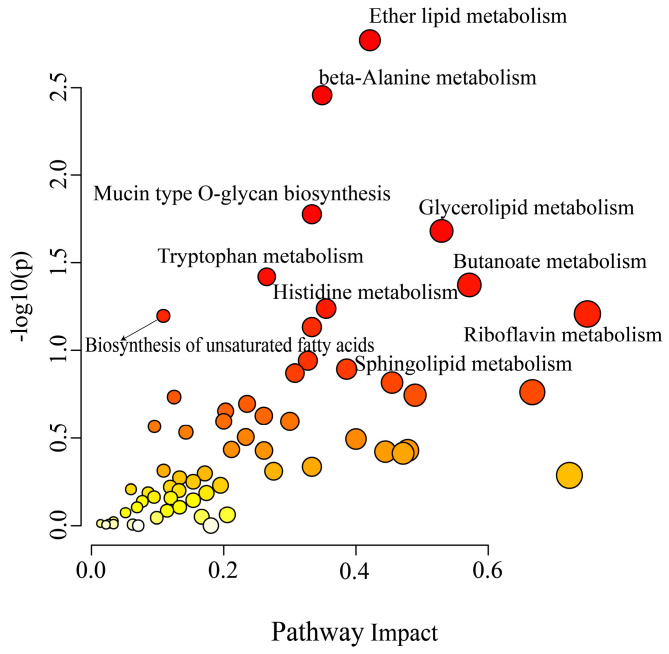
Effects of naringin on the metabolic pathways. The most impacted pathways are colored red according to the *p*-values generated by pathway enrichment analysis (*y*-axis) and pathway impact values from pathway topology analysis (*x*-axis).

There is increasing evidence that cellular metabolism plays a critical role in cardiovascular disease by regulating macrophage activation and function [19,20,21,22]. Thus, we further investigated the influence of naringin on the metabolic phenotypes of ox-LDL-stimulated THP-1 macrophages by metabolomic analysis. We identified 553 and 359 metabolites in positive and negative modes in the non-targeted metabolomic profiling. A significant difference was observed between the two groups in the PCA score plots, and the pooled quality control (QC) samples clustered together in both positive and negative ion modes, indicating the high stability and reproducibility of the LC-MS analysis (Figure 6A and Figure 7A). Furthermore, the PLS-DA models were created based on the detected metabolites to examine the differences between the naringin-treated samples and the controls, which exhibited apparent intra-group clustering and inter-group separations, indicating apparent metabolic alterations induced by naringin (Figure 6B and Figure 7B). Then, the metabolites with VIP > 1 and *p* < 0.05 were identified as differentially expressed metabolites (DEMs) (Figure 6C and Figure 7C). As a result, 50 and 38 DEMs were screened out in positive and negative modes, respectively, which exhibited differential enrichments in the naringin-treated samples and the controls (Figure 6D–F and Figure 7D–F). The results showed that naringin significantly affects lipid metabolism in macrophages, in particular, significantly increasing the levels of free fatty acids; acylcarnitines, which are necessary for fatty acid uptake into the mitochondria for β-oxidation; phosphatidylcholine (PC); phosphatidylethanolamine (PE); phosphatidylglycerol (PG); lysophosphatidylcholine (LPC); and lysophosphatidylethanolamine (LPE). To further explore the effect of naringin on macrophage metabolic phenotypes, joint pathway analysis of the transcriptomics and metabolomics data was performed by using MetaboAnalyst 5.0 (https://www.metaboanalyst.ca/, accessed on 13 January 2024), and the results showed that the DEGs and DEMs were enriched in metabolic pathways, including ether lipid metabolism, beta-alanine metabolism, glycerolipid metabolism, mucin type O-glycan biosynthesis, tryptophan metabolism, butanoate metabolism, histidine metabolism, riboflavin metabolism, the biosynthesis of unsaturated fatty acids and sphingolipid metabolism (Figure 8). Taken together, these results indicated that naringin rewired the metabolic pathways, especially promoting the lipid metabolism of macrophages, which was in line with the transcriptome results.

## 4. Discussion

AS is the major potential pathology of CVDs, which are the leading cause of morbidity and mortality worldwide [23]. There is increasing interest in the potential of natural products for the prevention and treatment of AS. Naringin, a citrus flavonoid abundant in citrus fruits, has significant therapeutic potential in the treatment or prevention of AS due to its antioxidative and anti-inflammatory actions [24,25]. Although naringin has been reported to exert an antiatherogenic function [26], its pharmacological mechanism is unclear. Aberrant lipid metabolism and inflammation are major contributors to foam cell formation during the development of AS [27]. Targeting foam cell formation was considered as a reasonable measure to protect against AS progression [28].

In this study, we comprehensively evaluated the effect of naringin on foam cell formation in THP-1 and RAW264.7 macrophages which, were induced by ox-LDL, and confirmed that naringin could inhibit foam cell formation and reduce intracellular lipid aggregation at a concentration of 4 µg/mL to 32 µg/mL. Furthermore, naringin significantly reduced TC and CE/TC and increased FC content in THP-1 and RAW.264.7 macrophages, indicating that naringin may exert its anti-AS effects by inhibiting the synthesis of cholesterol esters in macrophages. Excessive cholesterol uptake and decreased efflux can lead to foam cell formation in macrophages, suggesting that the homeostasis of cholesterol plays a very important role in AS development [27]. As expected, we found that naringin markedly inhibited the uptake of Dil-ox-LDL and promoted cholesterol efflux to maintain cholesterol homeostasis mechanistically through the downregulation of the key genes *MSR1* and *CD36* for lipid uptake and the upregulation of *ABCA1*, *ABCG1* and *SR-B1* responsible for cholesterol efflux. Consistently, Feng Wang et al. also confirmed that naringin can upregulate the hepatic expression of ABCA1 and SR-B1 in ApoE^−/−^ mice [29]. Additionally, the RNA-seq data showed that naringin significantly downregulated cholesterol synthesis-related genes such as *NR1H3*, *ANGPTL4*, *INSIG1*, *CYP51A1 and CYP27A1*, while it upregulated the genes *CYP46A1* and *OSBPL6* involved in the cholesterol metabolism pathway (Figure 5). Overall, these findings demonstrated that naringin inhibited foam cell formation by reducing lipid influx and promoting cholesterol efflux and metabolism in macrophages.

It is recognized that inflammation assumes a pivotal role in the formation of foaming cells, and lipid deposition may trigger inflammation, which, in turn, facilitates the adhesion and infiltration of monocytes, and eventually exacerbates foaming cell formation. Adhesion molecules (ICAM-1 and VCAM-1) and MCP-1/CCL2 promote the recruitment of monocytes to the endothelial layer and infiltration, ultimately leading to the accumulation of macrophage foam cells [30,31]. Our study shows that naringin markedly decreased the ox-LDL-induced increase in the protein and mRNA levels of ICAM-1, VCAM-1 and MCP-1 (Figure 4). The proinflammatory cytokines IL-1β, IL-6 and TNF-α, which serve as markers of inflammatory status, were also decreased by naringin, while the anti-inflammatory cytokine IL-10 was increased by naringin. Simultaneously, naringin also markedly reduced the levels of proinflammatory cytokines (MCP-1, IL-1β, IL-6 and TNF-α) in the medium (Figure 4C). This finding is in line with a previous report showing that naringin attenuated elevated inflammatory markers in LPS-induced jejunal barrier function in mice [32]. Accordingly, in this study, the comparative transcriptome analysis revealed that the inflammatory response signaling pathways (such as cytokine–cytokine receptor interaction, the MAPK signaling pathway and the TNF signaling pathway) were disrupted by naringin, which significantly downregulated most of the pro-inflammatory and chemokine-related genes (Figure 5D,E). Taken together, the powerful inhibitory effect of naringin on the ox-LDL-induced activation of the macrophage inflammatory response may make it capable of inhibiting foam cell formation and reversing the adverse factors of AS.

A number of studies have demonstrated that metabolic state affects macrophage phenotype and function [33]. It is currently appreciated that the inflammatory activation of macrophages is accompanied by increased glycolysis, which is considered as a determinant of inflammation in atherosclerosis [34]. Our study shows that most glycolysis-related genes were significantly reduced by naringin, especially the key genes in glycolysis, such as *HK3* and *PFKFB4* (Figure 6F). Additionally, it was reported that the glycolysis-related genes *HK2* and *PFKFB3* were highly expressed in monocytes from AS patients [35], and were also found in LPS-treated macrophages [18]. Specifically, the synthesis and oxidation of intracellular fatty acids regulate the inflammatory output of macrophages, in which fatty acid oxidation metabolism is linked to anti-inflammatory activity, while fatty acid synthesis is associated with a pro-inflammatory phenotype [19,34]. Interestingly, our study revealed that naringin reprogrammed lipid metabolism in macrophages by reducing the expression of triglycerides and fatty acid biosynthesis-related genes while upregulating fatty acid catabolism genes. Meanwhile, the acylcarnitines required for fatty acid oxidation were also significantly increased by naringin. Overall, we speculated that naringin may reprogram the metabolic phenotypes of macrophages by suppressing glycolysis and promoting lipid oxidation metabolism to restore macrophage phenotypes and functions.

It is noteworthy that although most studies have shown that naringin has a good safety profile it was observed in clinical trials that single oral doses of 2 g of naringin have no adverse effects on patients [11]), there are also studies reporting that naringin and naringenin are a constituents of grapefruit and the excessive intake of this fruit can lead to a syndrome of pseudo-hyperaldosteronism due to the blocking of the enzyme 11HSD2 [36,37]. Therefore, the safety and safe dosage range of naringin for clinical use still need further research.

## 5. Conclusions

In summary, our research has outlined potential mechanisms for naringin supplementation in inhibiting macrophage foam cell formation, which might offer new therapeutic approaches for AS. However, our study remains somewhat limited, and the findings need to be further validated in animal models. In addition, the direct targets of naringin and its specific regulatory pathways still need to be further explored. Further efforts are therefore required to elucidate the exact underlying mechanism of naringin in the pathological development of atherosclerosis.

## Figures and Tables

**Table 1:** Primer sequences for RT-qPCR analysis.

Genes	Forward	Reverse
*β-Actin*	5′-CACCATTGGCAATGAGCGGTTC-3′	5′-AGGTCTTTGCGGATGTCCACGT-3′
*ICAM-1*	5′-AGCGGCTGACGTGTGCAGTAAT-3′	5′-TCTGAGACCTCTGGCTTCGTCA-3′
*VCAM-1*	5′-GATTCTGTGCCCACAGTAAGGC-3′	5′-TGGTCACAGAGCCACCTTCTTG-3′
*MCP-1*	5′-AGAATCACCAGCAGCAAGTGTCC-3′	5′-TCCTGAACCCACTTCTGCTTGG-3′
*IL-6*	5′-AGACAGCCACTCACCTCTTCAG-3′	5′-TTCTGCCAGTGCCTCTTTGCTG-3′
*IL-10*	5′-TCTCCGAGATGCCTTCAGCAGA-3′	5′-TCAGACAAGGCTTGGCAACCCA-3′

## Data Availability

The original contributions presented in the study are included in the article, further inquiries can be directed to the corresponding author.
